# Inhibition of avian-origin influenza A(H7N9) virus by the novel cap-dependent endonuclease inhibitor baloxavir marboxil

**DOI:** 10.1038/s41598-019-39683-4

**Published:** 2019-03-05

**Authors:** Keiichi Taniguchi, Yoshinori Ando, Haruaki Nobori, Shinsuke Toba, Takeshi Noshi, Masanori Kobayashi, Makoto Kawai, Ryu Yoshida, Akihiko Sato, Takao Shishido, Akira Naito, Keita Matsuno, Masatoshi Okamatsu, Yoshihiro Sakoda, Hiroshi Kida

**Affiliations:** 1 Shionogi & Co., Ltd., Osaka, Japan; 2 Department of Disease Control, Faculty of Veterinary Medicine, Hokkaido University, Sapporo, Japan; 3 Research Center for Zoonosis Control, Hokkaido University, Sapporo, Japan; 4 Global Station for Zoonosis Control, Global Institution for Collaborative Research and Education (GI-CoRE), Hokkaido University, Sapporo, Japan; 5 Present Address: Organization for Research and Community Development, Gifu University, Gifu, Japan

## Abstract

Human infections with avian-origin influenza A(H7N9) virus represent a serious threat to global health; however, treatment options are limited. Here, we show the inhibitory effects of baloxavir acid (BXA) and its prodrug baloxavir marboxil (BXM), a first-in-class cap-dependent endonuclease inhibitor, against A(H7N9), *in vitro* and *in vivo*. In cell culture, BXA at four nanomolar concentration achieved a 1.5–2.8 log reduction in virus titers of A(H7N9), including the NA-R292K mutant virus and highly pathogenic avian influenza viruses, whereas NA inhibitors or favipiravir required approximately 20-fold or higher concentrations to achieve the same levels of reduction. A(H7N9)-specific amino acid polymorphism at position 37, implicated in BXA binding to the PA endonuclease domain, did not impact on BXA susceptibility. In mice, oral administration of BXM at 5 and 50 mg/kg twice a day for 5 days completely protected from a lethal A/Anhui/1/2013 (H7N9) challenge, and reduced virus titers more than 2–3 log in the lungs. Furthermore, the potent therapeutic effects of BXM in mice were still observed when a higher virus dose was administered or treatment was delayed up to 48 hours post infection. These findings support further investigation of BXM for A(H7N9) treatment in humans.

## Introduction

Influenza pandemics arise when novel reassortant influenza A viruses acquire zoonotic potential and adapt to enable efficient and widespread human-to-human infection^1^. The avian influenza A(H7N9) virus is an urgent concern for global public health due to the potential for pandemic spread^2^. In March 2013, the first outbreak in humans with an Asian lineage A(H7N9) were reported in China^3^. A(H7N9) was initially characterized as a low pathogenic avian influenza virus which caused mild disease in avian hosts^4^, however, of particular concern, were the acquisition of certain mutations (Leu226 in HA and Glu627 in PB2) which facilitated a switch to human-type receptor specificity enabling replication at temperatures present in the human respiratory tract^5–7^. Since early 2013, influenza A(H7N9) viruses have caused five distinct epidemic waves^8^, and laboratory-confirmed human cases admitted to hospital with A(H7N9) exhibited high case fatality rates^9^. The highest number of human cases with A(H7N9) occurred in China from October 2016 and the number of cases exceeded those reported in the previous waves^10,11^. Importantly, highly pathogenic A(H7N9) viruses were isolated from humans during the fifth epidemic wave^10^. As of October 2018, a total of 1,567 laboratory-confirmed human cases (with 615 deaths) of A(H7N9) infections in humans have been reported^12^.

Antiviral medications are of critical importance to reduce the burden of disease caused by A(H7N9) and the U.S. Centers for Disease Control and Prevention (CDC) recommends the use of antivirals for treatment of all hospitalized cases of human infection with novel influenza A viruses associated with severe disease^13^. While the A(H7N9) viruses are resistant to M2-ion channel blockers, treatment with oseltamivir and peramivir have been reported to be effective in patients with A(H7N9) infection^5,14^, with oseltamivir the most widely used drug^15^. Importantly, evidence for the therapeutic effects of oseltamivir treatment has been accumulating; however, the available NAIs show limited ability to reduce viral titers, therefore, there may be limited virologic or therapeutic advantages, especially in patients with severe influenza with high viral loads^16–18^. In addition, the emergence of drug resistant mutants have been detected after NAI treatment against seasonal and zoonotic influenza A virus infections^19–23^. In particular, the arginine to lysine mutation at position 292 (N2 numbering) in the NA (NA-R292K) confers a reduced susceptibility to all approved NAIs, and has been identified in A(H7N9) clinical isolates, such as A/Shanghai/1/2013 and A/Taiwan/1/2013^24,25^. Therefore, novel antivirals that overcome the current limitations for treatment of A(H7N9) infection are urgently required to decrease both morbidity and mortality in infected subjects and to assist in the preparedness to help mitigate widespread transmission in possible future pandemics.

The influenza RNA polymerase complex, composed of the PA, PB1 and PB2 subunits, helps mediates a unique “cap-snatching” mechanism^26^ and thereby plays a crucial role during both the replication and transcription stages of the viral life cycle. As a result the cap-snatching mechanism is considered a promising antiviral target^27^. The cap-dependent endonuclease (CEN) in the PA subunit cleaves bound-capped RNAs 10–13 nucleotides from the 5′ ends of nascent transcripts to generate a primer for the synthesis of viral mRNA. The active center of CEN is also highly conserved across seasonal, pandemic, and highly pathogenic avian influenza viruses^26,28^, indicating that CEN inhibitors have the potential to be broadly-active anti-influenza drugs^28–30^.

Baloxavir marboxil (BXM), which is converted metabolically to its active form baloxavir acid (BXA), is an orally available CEN inhibitor that has recently been approved for clinical use (single dose of 40 mg for patients 40 kg to <80 kg or 80 mg for patients ≥ 80 kg) in adults and adolescents in Japan and the United States^31–33^. To enhance oral absorption of BXA, the phenolic hydroxyl group was modified, yielding the prodrug BXM. Notably, BXA exhibits broad antiviral activities against several subtypes of influenza A and B viruses *in vitro*, and co-crystal structures of PA endonuclease domain from influenza A and B viruses support broad spectrum activity for this class of compounds^32,34,35^. Therefore, BXM is anticipated to be an additional and alternative option for treatment of avian-origin influenza A viruses; however, information on the *in vitro* potency of BXA and *in vivo* efficacy of BXM is still limited. In the present study, we report the antiviral activities of BXA and BXM against human influenza A(H7N9) viruses, including highly pathogenic avian influenza viruses. Our findings support further investigation of the therapeutic efficacy of BXM treatment in A(H7N9)-infected patients.

## Results

### Inhibitory effect of BXA on A(H7N9) virus replication *in vitro*

Avian-origin influenza A(H7N9) viruses typically harbor a polymorphic alanine to serine substitution at residue 37 in the PA (A37S)^36,37^, which is involved in BXA binding in the endonuclease domain (Table 1)^32,38^. To examine whether BXA possessed inhibitory activity against human A(H7N9) virus *in vitro*, we selected strains from subtypes A(H7N9) and A (H7N3), including highly pathogenic avian influenza viruses, isolated from 2013 to 2018 harboring alanine or serine at residue 37 in the PA. To compare the degree of inhibition of virus replication by approved drugs, a yield reduction assay using Madin-Darby canine kidney (MDCK) cells was employed. The mean concentration achieving 90% (1-log) reduction in virus titer (EC_90_) values of approved drugs and virus titers under different drug concentrations are shown in Table 2 and Supplementary Table 1, respectively. BXA showed inhibitory activity against A/Anhui/1/2013 (H7N9) strain as previously reported^35^ and exhibited comparable potency against A/Anhui/1/2013 (H7N9) harboring the NA-R292K substitution to the wild-type, indicating no-cross resistance with NAIs was observed. BXA also exhibited comparable potency against H7 low and highly pathogenic avian influenza viruses to A/Anhui/1/2013 (H7N9) strain. Notably, BXA at four nanomolar concentration achieved a 1.5–2.8 log reduction in viral titers (Supplementary Table 1). By contrast, NAIs or the RNA-dependent RNA polymerase inhibitor favipiravir required approximately 20-fold or higher concentrations to achieve the same levels of virus reduction as BXA. These results suggest that BXA has high antiviral activity against A(H7N9) despite the viruses possessing the polymorphic PA-A37S substitution located in the adjacent BXA-binding site. In addition, BXA exhibits a 20-fold greater degree of inhibition of virus replication compared to the other approved drugs *in vitro*.

**Table 1:** Amino acid polymorphisms of the BXA binding domain in PA from human and avian influenza A viruses.

Influenza virus strain	Subtype	PA amino acid position^a^												
		20	23	24	34	37	38	41	80	108	119	130	134	199
A/Anhui/1/2013	H7N9	A	E	Y	K	**S**	I	H	E	D	E	Y	K	E
A/duck/Japan/AQ-HE28-3/2016	H7N9	A	E	Y	K	**S**	I	H	E	D	E	Y	K	E
A/duck/Japan/AQ-HE29-22/2017^b^	H7N9	**T**	E	Y	K	**S**	I	H	E	D	E	Y	K	E
A/duck/Japan/AQ-HE30-1/2018^b^	H7N3	A	E	Y	K	A	I	H	E	D	E	Y	K	E
Human influenza viruses isolated (As of October 24, 2018)^c^	H7N9	A (91.5)	E (100)	Y (100)	K (100)	**S** (99.9)	I (100)	H (100)	E (100)	D (100)	E (100)	Y (100)	K (100)	E (99.8)
	H1N1	A (99.3)	E (100)	Y (99.9)	K (100)	A (100)	I (99.9)	H (100)	E (100)	D (100)	E (100)	Y (100)	K (100)	E (100)
	H3N2	A (98.3)	E (100)	Y (100)	K (100)	A (100)	I (100)	H (100)	E (100)	D (100)	E (100)	Y (100)	K (100)	E (100)
	H5N1	A (85.2)	E (100)	Y (100)	K (100)	A (100)	I (100)	H (100)	E (100)	D (100)	E (100)	Y (100)	K (100)	E (100)

^a^The indicated amino acids have been previously shown to be involved in BXA binding to the active center of the endonuclease domain in the PA subunit (residues 20, 24, 34, 37, 38, 41, 80, 108, 119, 130, and 134) and associated with reduced susceptibility to BXA (residues 23, 37, 38 and 199) as reported previously^32^. The amino acids different from the consensus sequence of human influenza A viruses are highlighted in boldface and underlined. ^b^Highly pathogenic avian influenza viruses. ^c^The consensus sequences were determined by alignment analysis with the full-length PA sequences obtained from the National Center for Biotechnology Information (NCBI) and Global Initiative on Sharing All Influenza Data (GISAID) on October 24, 2018. Each number shown in parentheses represents frequency (%) of the most frequent variants among 1,094, 10,312, 13,185 and 196 of PA sequences from H7N9, H1N1, H3N2 and H5N1, respectively.

**Table 2:** Antiviral activities of BXA and reference compounds against human and avian A(H7N9) viruses in a yield reduction assay in MDCK cells.

Influenza virus strain	Mean EC_90_ (nM) ± SD				
	Baloxavir acid	Oseltamivir acid	Zanamivir hydrate	Laninamivir	Favipiravir
A/Anhui/1/2013 (H7N9)	0.79 ± 0.35	12.90 ± 4.78	22.71 ± 6.42	11.58 ± 2.62	18,454.53 ± 14,157.33
A/Anhui/1/2013 NA-R292K (H7N9)^a^	1.12 ± 0.53	142,389.79 ± 6,601.02	NT ^c^	NT ^c^	17,025.75 ± 4,221.17
A/duck/Japan/AQ-HE28-3/2016 (H7N9)	0.62 ± 0.11	38.52 ± 18.19	NT ^c^	NT ^c^	12,014.40 ± 7,990.32
A/duck/Japan/AQ-HE29-22/2017 (H7N9)^b^	0.69 ± 0.29	12.89 ± 9.85	NT ^c^	NT ^c^	17,191.98 ± 13,933.15
A/duck/Japan/AQ-HE30-1/2018 (H7N3)^b^	1.13 ± 0.58	27.86 ± 17.98	NT ^c^	NT ^c^	18,879.83 ± 13,666.58

^a^Virus generated by reverse genetics. ^b^Highly pathogenic avian influenza virus. ^c^Not tested. Data represent mean ± standard deviation from three independent experiments in MDCK cells.

### Protective efficacy of BXM on lethal infections of A(H7N9) *in vivo*

**Figure 1:**
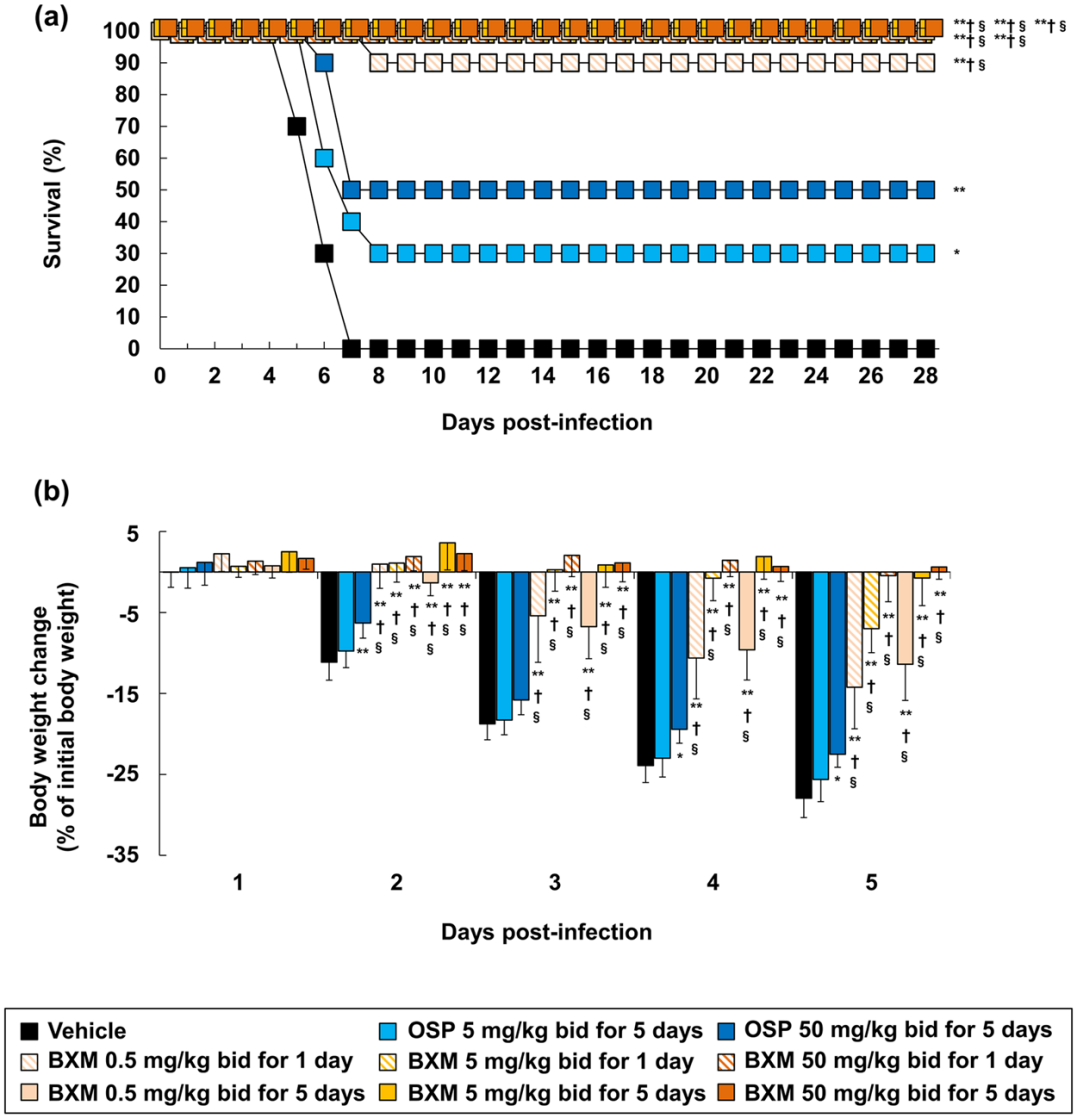
Therapeutic effects of BXM on survival and weight loss in a lethal infection model of mice infected with a low dose of the A(H7N9) virus. Mice were intranasally inoculated with 4.0 × 10^5^ TCID_50_/mouse (10.4 MLD_50_) of A/Anhui/1/2013 (H7N9) viruses, and treatment was started immediately after virus inoculation (n = 10/group). Survival time (**a**) and body weight loss (**b**) were monitored through a 28-day period after the infection. bid (bis in die): twice a day. The log-rank test was applied for comparison of the survival time between each group (*p < 0.05, **p < 0.001 compared to vehicle, ^†^*p* < 0.01 compared to OSP at 5 mg/kg twice a day, ^§^*p* < 0.05 compared to OSP at 50 mg/kg twice a day). Dunnett’s multiple-comparison method was applied for statistical analysis of body weight changes (*p < 0.01, **p < 0.001 compared to vehicle, ^†^*p* < 0.001 compared to OSP at 5 mg/kg twice a day, ^§^*p* < 0.001 compared to OSP at 50 mg/kg twice a day). The body weights at 5 dpi were calculated from nine mice in vehicle-treated group because one of the ten mice showed more than 30% reduction and was euthanized.

In order to evaluate the effects of BXM against A(H7N9) in a lethal infection model, mice were inoculated with 10.4 times of 50% mouse lethal dose (MLD_50_) of A/Anhui/1/2013 (H7N9). All vehicle-treated mice died within 7 days post-infection (dpi) and mean day to death was 6 days (Fig. 1a). Clinically-equivalent dosing of oseltamivir phosphate (OSP), 5 mg/kg twice a day for 5 days^39^, and a supratherapeutic dose, 50 mg/kg twice a day for 5 days, resulted in 30% and 50% survival, respectively. In this setting, survival rates of BXM at 0.5, 5, and 50 mg/kg twice a day for 1 day were 90%, 100% and 100%, respectively. When compared to the survival time at 28 dpi, all groups treated with BXM showed significant prolonged survival times compared with the groups administered with vehicle or OSP. Dramatic body weight loss after infection was observed in the vehicle-treated control group and reached a 28% decrease at 5 dpi (Fig. 1b and Supplementary Fig. 2). When treated with OSP at 5 mg/kg twice a day for 5 days, body weight change was comparable to that of the vehicle-treated group, whereas OSP treatment at 50 mg/kg twice a day for 5 days significantly suppressed body weight loss. By contrast, BXM significantly prevented body weight loss from day 2 to 5 in a dose-dependent manner, when compared to vehicle and OSP. These results indicate that BXM exerts improvements in survival in mice infected with A/Anhui/1/2013 (H7N9).

### Effects of BXM on virus titers in mice infected with A(H7N9)

**Figure 2:**
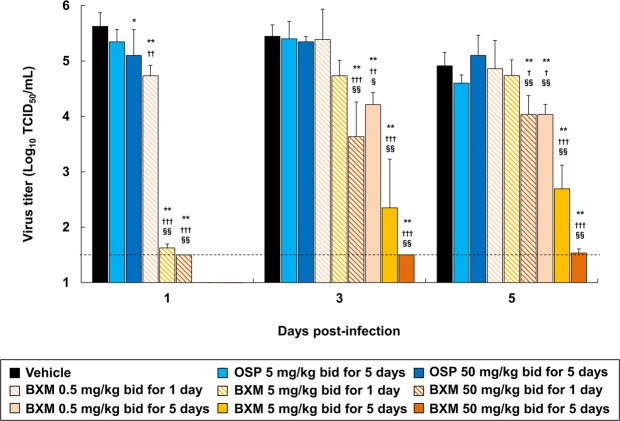
Inhibitory effect of BXM on virus titers in the lungs of mice infected with a low dose of the A(H7N9) virus. Mice were inoculated with 4.0 × 10^5^ TCID_50_/mouse (10.4 MLD_50_) of A/Anhui/1/2013 (H7N9) virus and treatment was started immediately after virus inoculation (n = 5/group). The virus titers (TCID_50_) in lungs of mice at 1, 3 and 5 dpi were measured in MDCK cells. The lower limit of quantification of the virus titer is indicated by a dotted line (1.5 Log_10_ TCID_50_/mL). Dunnett’s multiple-comparison method was conducted for statistical comparison (*p < 0.05, **p < 0.001 compared to vehicle, ^†^*p* < 0.05, ^††^*p* < 0.01, ^†††^*p* < 0.001 compared to OSP at 5 mg/kg twice a day, ^§^*p* < 0.01, ^§§^*p* < 0.001 compared to OSP at 50 mg/kg twice a day).

In order to examine the inhibitory effects of BXM on the viral replication of A/Anhui/1/2013 (H7N9) *in vivo*, virus titers in lung homogenates derived from infected mice were measured at 1, 3, and 5 dpi. BXM treatment at 5 and 50 mg/kg twice a day decreased virus titers in the lungs of mice by more than 3-logs compared to that of vehicle- or OSP-treatment group, while virus titers of all OSP-treated groups were comparable to those of vehicle-treated group at 1 dpi (Fig. 2). Although gradual increases of virus titers for 1-day dosing of BXM group were observed after withdrawal of treatment, virus titers were suppressed by more than 2 or 3-logs following repeated BXM treatment at 5 and 50 mg/kg compared to that of vehicle- or OSP-treatment groups. Additionally, mutation analysis of the PA N-terminal domain (residues 1 to 209) of A/Anhui/1/2013 (H7N9) was performed on lung homogenates of the infected mice treated with BXM^26^. We found no amino acid changes in the analyzed regions including residues implicated in BXA resistance by affecting BXA binding to the active center of the endonuclease domain, such as isoleucine at position 38 in the PA (Ile38)^32,33^. These results suggest that BXM has profound inhibitory effects on viral replication in the lungs of mice infected with A(H7N9).

### Prevention of proinflammatory cytokines and chemokines production in the lungs of mice following BXM treatment

**Figure 3:**
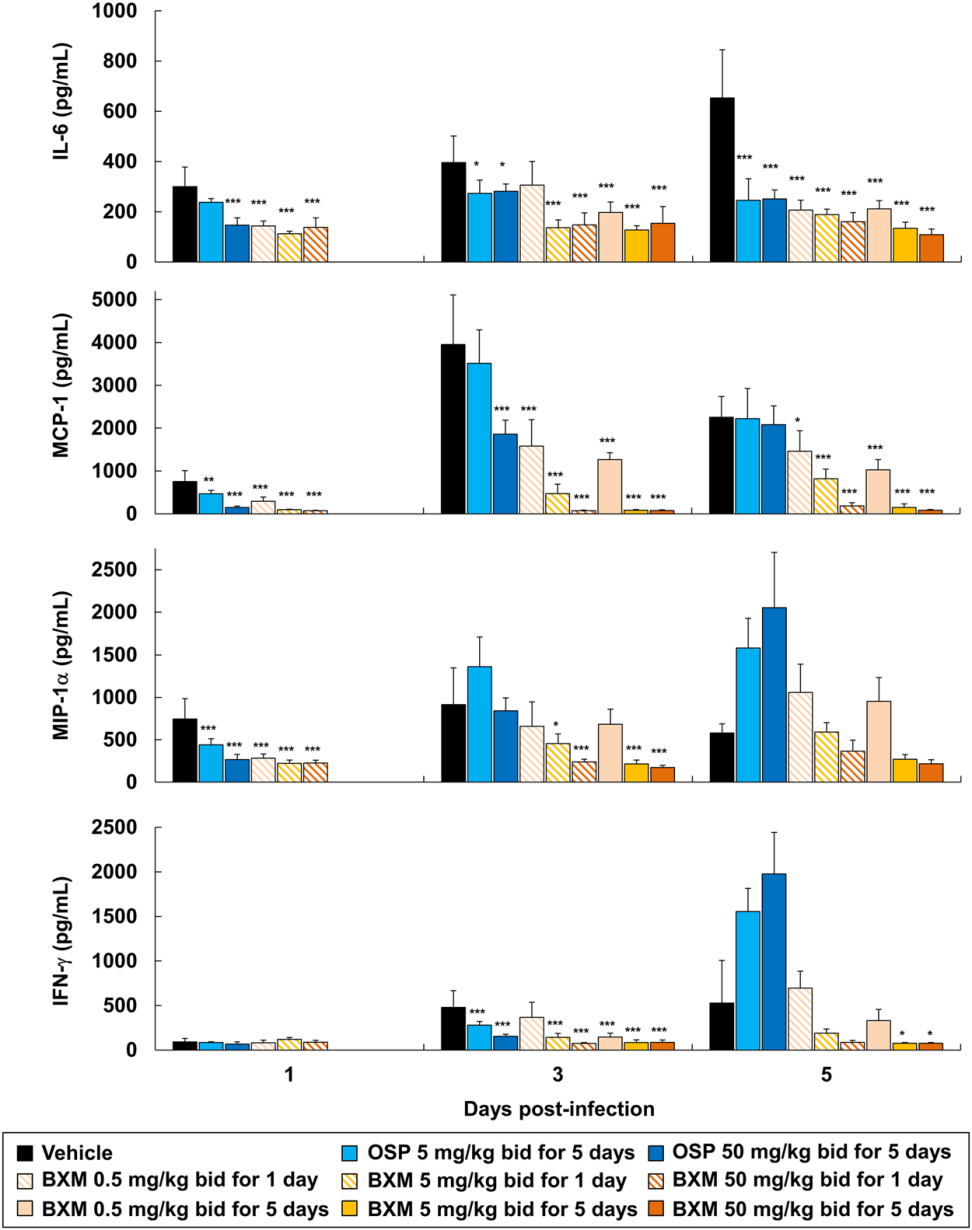
Suppressive effects of BXM on proinflammatory cytokine and chemokine production in the lungs of mice infected with a low dose of the A(H7N9) virus. Mice were intranasally infected with 4.0 × 10^5^ TCID_50_/mouse (10.4 MLD_50_) of A/Anhui/1/2013 (H7N9) virus, and treatment was started immediately after virus inoculation (n = 5/group). The proinflammatory cytokines and chemokines, IL-6, MCP-1, MIP-1α and IFN-γ, in the lungs at 1, 3 and 5 dpi were quantified. Dunnett’s multiple-comparison method was employed for the comparison (*p < 0.05, **p < 0.01, ***p < 0.001 compared to vehicle).

It has been previously reported that production of proinflammatory cytokines and chemokines, such as interleukin (IL)-6, monocyte chemoattractant protein (MCP)-1, macrophage inflammatory protein (MIP)-1α, and interferon (IFN)-γ, is markedly elevated in A(H7N9)-infected patients^40–42^. Furthermore, A(H7N9) infection-associated inflammation in the lungs resulted in a rapidly progressive pneumonia and development of acute respiratory distress syndrome in the majority of hospitalized patients^3,43^. Therefore, the impact of BXM treatment upon proinflammatory cytokines and chemokines production in the lung of mice inoculated with A/Anhui/1/2013 (H7N9) was evaluated. Treatment of the A(H7N9)-infected mice with BXM at 5 and 50 mg/kg twice a day for 1 and 5 days resulted in a significantly less pronounced production of both proinflammatory cytokines and chemokines when compared with vehicle-received mice, consistent with the reduction of virus titers in the lungs of mice (Fig. 3). In contrast, OSP treatment showed a limited inhibitory effect on proinflammatory cytokines and chemokines production in the lungs of mice, and increased MIP-1α and IFN-γ production at 5 dpi. These results demonstrate that BXM has a profound inhibitory effect on both proinflammatory cytokine and chemokine production in the lungs of A(H7N9)-infected mice.

### Protective effect of BXM on lethality in mice infected with high doses of A(H7N9)

**Figure 4:**
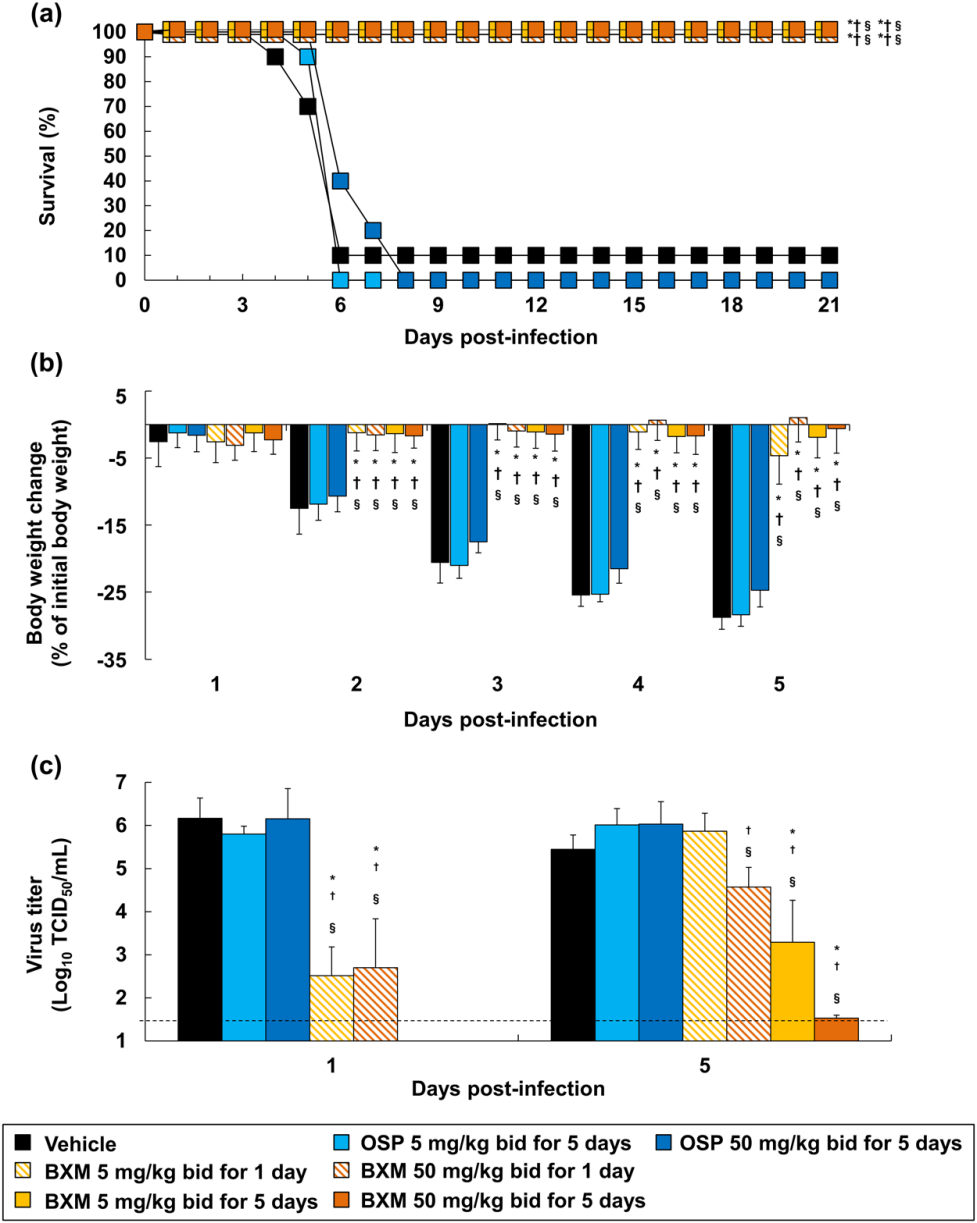
Effects of BXM on survival, weight loss and virus titers in the lungs in mice infected with a high dose of the A(H7N9) virus. Mice intranasally infected with 1.2 × 10^6^ TCID_50_/mouse (31.1 MLD_50_) of A/Anhui/1/2013 (H7N9) virus were administrated with treatment started immediately after virus inoculation, and survival time (**a**) and body weight loss (**b**) were analyzed. The log-rank test was applied for survival time (*p < 0.001 compared to vehicle, ^†^*p* < 0.001 compared to OSP at 5 mg/kg twice a day, ^§^*p* < 0.001 compared to OSP at 50 mg/kg twice a day). Dunnett’s multiple-comparison method was employed for body weight changes (*p < 0.001 compared to vehicle, ^†^*p* < 0.001 compared to OSP at 5 mg/kg twice a day, ^§^*p* < 0.001 compared to OSP at 50 mg/kg twice a day). Body weights at 4 and 5 dpi were calculated from nine mice because one of the ten mice in vehicle-treated group showed more than 30% reduction and was euthanized. (**c**) The virus titers (TCID_50_) in the lungs of mice at 1 and 5 dpi were measured. The lower limit of quantification of the virus titer is indicated by a dotted line (1.5 Log_10_ TCID_50_/mL). Dunnett’s multiple-comparison method was applied for the comparison (*p < 0.05, **p < 0.001 compared to vehicle, ^†^*p* < 0.05, ^††^*p* < 0.01, ^†††^*p* < 0.001 compared to OSP at 5 mg/kg twice a day, ^§^*p* < 0.01, ^§§^*p* < 0.001 compared to OSP at 50 mg/kg twice a day).

It has been described that the pathogenicity caused by A(H7N9) challenge in mice increased in a dose-dependent manner^5,44^, and therefore we next investigated the protective effect of BXM in a high-dose challenge model of A(H7N9) infection. Mice were inoculated with 31.1 MLD_50_ of A/Anhui/1/2013 (H7N9) and were then treated with OSP or BXM beginning immediately after virus inoculation. The mice administered with OSP at 5 and 50 mg/kg twice a day for 5 days died within 6 and 8 dpi, respectively, and no significant difference was observed on survival time up to 21 dpi compared to vehicle-treated group (Fig. 4a). Strikingly, even in these high dose challenge experimental conditions, BXM treatment at 5 and 50 mg/kg twice a day completely prevented mortality in all tested groups. BXM also significantly reduced the body weight loss in all the tested groups, whereas OSP treatment did not impact on body weight changes (Fig. 4b). Moreover, following 5-day dosing of BXM, body weight loss was within 5% throughout 28 days (Supplementary Fig. 3). In addition, BXM also significantly reduced virus titers in the lungs of mice, whereas OSP treatment had limited effect (Fig. 4c), consistent with the prior results from the 10.4 MLD_50_ infection model (Fig. 2). Overall, these results confirmed that BXM drastically reduces virus titers in the lungs of mice infected with A/Anhui/1/2013 (H7N9) at a high dose, resulting in greater efficacy than NAIs against lethal infection of A(H7N9) *in vivo*.

### Delayed treatment of BXM on lethal A(H7N9) infection

**Figure 5:**
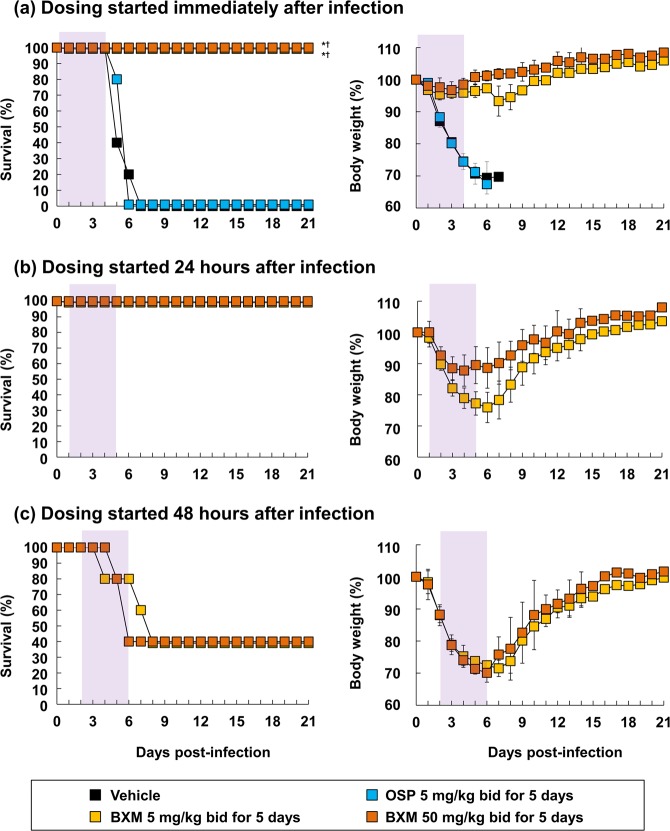
Effects of delayed treatment of BXM on a low dose of A(H7N9) infection. Mice were intranasally inoculated with 4.0 × 10^5^ TCID_50_/mouse (10.4 MLD_50_) of A/Anhui/1/2013 (H7N9) viruses, and BXM treatment was started at (**a**) immediately, (**b**) 24 or (**c**) 48 hours after virus inoculation (n = 5/group). Vehicle or OSP treatment was started immediately after virus inoculation (n = 5/group). Survival time and body weight loss were monitored through a 21-day period after the infection. The shaded area represents the treatment period. The log-rank test was applied for comparison of the survival time between each group (*p < 0.01 compared to vehicle, ^†^*p* < 0.01 compared to OSP at 5 mg/kg twice a day).

To further investigate the therapeutic effect of BXM, mice infected with 10.4 times of MLD_50_ of A/Anhui/1/2013 (H7N9) were treated with BXM starting after 24 and 48 hours after infection, and subsequently survival and body weight loss was monitored. Mice without treatment died 5 to 7 days after infection, consistent with findings presented in Fig. 1. In this experimental condition, the 24 hours delayed treatment of BXM at 5 and 50 mg/kg twice a day for 5 days resulted in complete protection against lethal infection with A/Anhui/1/2013 (H7N9) virus (Fig. 5). The therapeutic effect was still observed with the mice given BXM at 48 hours after infection. A time-dependent protective effect by means of body weight change was confirmed on all BXM-treated mice. These results suggest that BXM exhibits therapeutic effects against A/Anhui/1/2013 (H7N9) in mice when BXM treatment is delayed up to 48 hours after infection.

## Discussion

In this study, we evaluated the *in vitro* antiviral activity of BXA against influenza A(H7N9) viruses, including highly pathogenic avian influenza viruses, and the therapeutic effect of BXM *in vivo* in murine lethal models employing influenza A(H7N9) virus. In the yield reduction assays *in vitro*, BXA showed superior potency against replication of A/Anhui/1/2013 (H7N9) and recent isolates of highly pathogenic avian H7 viruses compared to the approved drugs. Given that the A/duck/Japan/AQ-HE28-3/2016 (H7N9) and A/duck/Japan/AQ-HE29-22/2017 (H7N9) strains based on PA gene segments are closely related phylogenetically to human A(H7N9) isolated in the fifth wave (Supplementary Fig. 1)^45^, BXA should exhibit inhibitory effect against clinical isolates of highly pathogenic A(H7N9). Furthermore, BXM protected from lethal infection and exhibited significant decreases in both virus replication and proinflammatory responses in mice infected with A/Anhui/1/2013 (H7N9). Further investigations, to study the inhibitory activity of BXA against recent isolates of A(H7N9), are warranted to examine further the utility of BXM.

Although NAI-treatment for A(H7N9) infection is effective and recommended, it was reported that NAI-resistant variants (e.g. NA-R292K) were isolated from human cases^24,25,46^ or NAI-resistant viruses have emerged during treatment^47,48^. In this study, A/Anhui/1/2013 (H7N9) bearing the R292K substitution in the NA protein conferred resistance to oseltamivir acid (Table 2), whereas the mutation does not impact upon BXA susceptibility, confirming that no cross-resistance relationship exists between NAI and BXA. On the other hand, it has been reported that BXM treatment occasionally induced emergence of viruses with reduced susceptibility to BXA, that harbor the glutamic acid to lysine or glycine at position 23, the alanine to threonine at position 37, the isoleucine to threonine, phenylalanine or methionine at position 38 and the glutamic acid to glycine at position 199 substitutions in PA protein^33,35^. Therefore, we carefully monitored emergence of the variant viruses throughout the experiments, but no substitutions in the PA N-terminal region (residues 1 to 209) were detected following BXM treatment in mice infected with A(H7N9). Given that A(H7N9) harbors the polymorphic A37S substitution adjacent to Ile38, there is a hypothetical possibility that A37S effects on the frequency of the detection of Ile38 substitutions. Hence, it would be worth undertaking resistance isolation experiments with A(H7N9) virus and analyzing the genetic compatibility of the viruses bearing both A37S and I38T substitutions.

The therapeutic effect of oral BXM administration was investigated following A(H7N9) challenge in a lethal infection murine model. In a previous report, 5 days dosing of OSP had limited effect on the inhibition of body weight loss and the concomitant reduction of virus titers in mice infected with A/Anhui/1/2013 (H7N9) strain^5^. Comparable results were obtained in this study. In our model, we showed that only 1-day dosing of BXM at 0.5 mg/kg twice a day were sufficient for significant reduction of virus titers in the lungs of mice. Furthermore, 5 or 50 mg/kg twice a day dosing of BXM achieved dramatic reductions in virus titers compared to the vehicle and OSP-treated groups and resulted in significant improvements in survival. Five-day dosing of BXM at 5 or 50 mg/kg twice a day also achieved dramatic reductions in virus titers and extended the treatment window, which indicated that repeating dose of BXM might be a reasonable option in severe cases of A(H7N9) infection. It has been suggested that the target plasma BXA concentration 24 h after a single-dose (C_24_) be set at 6.85 ng/mL in non-clinical and clinical studies^49–51^. The plasma concentration of BXA could be maintained above the target concentration of 6.85 ng/mL for at least 5 days following oral administration of BXM at 40 mg in humans^51^. Although it is difficult to set a clinically equivalent dose regimen in mice owing to the crucial difference in half-life of BXA in plasma after oral BXM administration between humans (85.9 hours at 40 mg BXM) and mice (2.24 to 3.14 hours at 0.5 to 50 mg/kg BXM), the C_24_ of BXA after 1-day dosing of BXM at 50 mg/kg twice a day, as well as C_120_ of BXA after 5-day dosing of BXM at 5 mg/kg twice a day in mice are expected to be lower than or close to those in humans^49–51^. Therefore, the dosages used in the mouse model of BXM at 50 mg/kg twice a day for 1 day or at 5 mg/kg twice a day for 5 days are comparable to the clinical dosages. On the other hand, a C_120_ of BXA after 5-day dosing of BXM at 50 mg/kg twice a day may be higher than that in humans. The existing evidence strongly suggests that BXM is has superior protective efficacy against A(H7N9) infection in murine models when compared to OSP, although further pharmacokinetic and pharmacodynamic analyses in mice and humans are required for a more complete understanding of these increased therapeutic effects exerted by BXM.

Tsang and co-workers reported that patients with high viral load correlated with the severity of influenza symptoms^52^. It has been shown that the potency of oseltamivir acid was diminished at higher multiplicities of infection (MOI) in MDCK cells in an MOI-dependent manner^53^, and OSP exhibited lower efficacy against higher infectious doses in a ferret model of influenza A(H5N1) virus infection^54^. Consistent with these observations, we confirmed that OSP did not reduce either morbidity or mortality in mice infected with high infectious doses of A(H7N9). We can infer from these findings that the therapeutic efficacy of OSP against influenza A virus infection attenuates dependent on the infectious dose. It is therefore particularly noteworthy that we demonstrated that 1-day dosing of BXM at 5 or 50 mg/kg twice a day showed greater efficacy against high dose A(H7N9) infection when compared to NAI OSP. Notably, favipiravir, which targets the viral RNA-dependent RNA polymerase, also exerted a strong effect upon the reduction of virus titers in the lungs of mice infected with high titers A(H7N9) when compared to NAIs^5^. These results suggest that inhibition of the viral RNA polymerase machinery, particularly the “cap-snatching” mechanism, may have the potential to be more effective for reducing virus titers and provide improved benefits for the treatment of severe influenza infections with high viral burdens.

In human infection with A(H7N9), virus-induced proinflammatory cytokine and chemokine dysregulation in the lungs or serum contributes to disease severity^55,56^. In addition, high levels of proinflammatory cytokines/chemokines were produced in the lungs of mice and cynomolgus macaques infected with A(H7N9) clinical isolates that replicated efficiently in the lungs^5,57^. In a murine model, suppression of proinflammatory cytokine/chemokine production positively correlated with both a reduction of virus titers in lungs and the disease severity^58^. Proinflammatory cytokines and chemokines are highly induced in the early phase of influenza virus infection and are associated with airway inflammation^56,59^; thus, BXM may serve to ameliorate severe influenza pneumonia due to exerting an inhibitory effect upon the production of IL-6, MCP-1 and MIP-1α in the early stages of virus infection. Here we have shown that IFN-γ production in the lungs was also suppressed by treatment with BXM. IFN-γ production in macrophages during influenza virus infection is thought to be critically important in sustaining the cytokine/chemokine storm, and IFN-γ acts to upregulate CXCL10 transcription in airway epithelial cells, which result in infiltration of effector T cells to the lung airways^60,61^. Reduction of IFN-γ thus likely limits cytokine overproduction in the lungs. These results suggest that BXM prevented virus replication followed by a concomitant decrease in the production of proinflammatory cytokines and chemokines. Thus, inhibition of virus replication in the early stages of infection appears therefore to be critically important for the amelioration of host dysfunction which likely serves to mediate the therapeutic effects.

In conclusion, this study demonstrates the high potency of BXA and BXM against influenza A(H7N9) viruses *in vitro* and *in vivo* compared to other currently approved antivirals. Although further investigations are required to clarify therapeutic effects against highly pathogenic avian-origin influenza A viruses *in vivo*, the existing evidence supports and warrants the consideration of BXM as an alternative therapeutic option for the treatment of A(H7N9) infection in humans.

## Methods

### Compounds

Baloxavir marboxil (BXM) and baloxavir acid (BXA) were synthesized at Shionogi & Co., Ltd.. Oseltamivir acid, laninamivir were purchased from Toronto Research Chemicals Inc. (Toronto, Ontario, Canada). Oseltamivir phosphate and zanamivir hydrate were obtained from Sequoia Research Products Ltd. (Pangbourne, UK). Favipiravir was supplied by PharmaBlock Sciences, Inc. (Nanjing, China).

### Cells and viruses

The Madin-Darby canine kidney (MDCK; European Collection of Cell Cultures) cells were maintained at 37 °C under 5% CO_2_ in minimum essential medium (MEM; Nissui Pharmaceutical) supplemented with 10% heat-inactivated fetal bovine serum, 2 mmol/L L-glutamine, 50 units/mL penicillin, 50 µg/mL streptomycin and 0.05% sodium hydrogen carbonate. The non-mouse adapted influenza A/Anhui/1/2013 (H7N9) virus, which is a clinical isolate and is pathogenic in mice^41^, A/duck/Japan/AQ-HE28-3/2016 (H7N9), A/duck/Japan/AQ-HE29-22/2017 (H7N9) and A/duck/Japan/AQ-HE30-1/2018 (H7N3) were propagated in embryonated chicken eggs and harvested from virus-containing allantoic fluids. A/Anhui/1/2013 (H7N9) and A/duck/Japan/AQ-HE28-3/2016 (H7N9) possess low pathogenicity, A/duck/Japan/AQ-HE29-22/2017 (H7N9) and A/duck/Japan/AQ-HE30-1/2018 (H7N3) possess high pathogenicity^45^. Recombinant A/Anhui/1/2013 (H7N9) virus harboring NA-R292K was generated by plasmid-based reverse genetics^62^. Recombinant virus was propagated in embryonated chicken eggs and harvested from virus-containing allantoic fluids. Infectious titers were determined by standard 50% tissue culture infectious dose (TCID_50_) assay in MDCK cells.

### Virus yield reduction assay

Two days prior to infection, MDCK cells were seeded in 96-well plates and the cells were infected with each virus at 100 TCID_50_/well. The infected cells were incubated at 35 °C under 5% CO_2_ for 1 hour and wash out the virus inoculum, followed by addition of the fresh medium including 2.5 µg/ml trypsin and defined concentrations of test compounds. BXA and favipiravir were dissolved in dimethyl sulfoxide and NAIs were dissolved in distilled water. The cells were incubated at 35 °C under 5% CO_2_ for 24 hours and virus titers (TCID_50_/mL) in the culture supernatants were determined in MDCK cells. The 90% effective concentration (EC_90_) was calculated as the concentration decreasing the virus titers in the culture supernatant to 10% of untreated control values by the linear interpolation method.

### Genetic analysis

PA sequences of 10,312 clinical isolates for A(H1N1), 13,185 for A(H3N2), 196 for A(H5N1) and 1,094 for A(H7N9), were downloaded from the National Center for Biotechnology Information (NCBI) and Global Initiative on Sharing All Influenza Data (GISAID) on October 24, 2018. Amino acid sequences of the PA protein were aligned by the ClustalW program in the component of Pipeline Pilot 2018 (BIOVIA), and conservation of amino acid residues in close proximity to the BXA was calculated within individual influenza virus subtypes. The nucleotide sequences were phylogenetically analyzed based on PA genes of H7 avian influenza viruses by the maximum-likelihood method with a Tamura–Nei model and bootstrap analysis (n = 1000) using MEGA 7.0 software^63^ with default parameters. Sequence data of PA genes obtained in the present study were compared with those of other reference strains presented in a previous report^45^.

### Animal experiments

#### Experiment 1

Six-week-old female BALB/c mice (Japan SLC, Inc.) were maintained under a controlled temperature environment and humidity. Under anesthesia (1.6 mg/mL zolazepam hydrochloride, 1.6 mg/mL tiletamine hydrochloride and 1.9 mg/mL xylazine hydrochloride in saline), mice were infected intranasally (50 µL/mouse) with 4.0 × 10^5^ TCID_50_ (low dose, 10.4 of 50% mouse lethal dose [MLD_50_]) or 1.2 × 10^6^ TCID_50_ (high dose, 31.1 MLD_50_) of A/Anhui/1/2013 (H7N9) virus. On the morning of the first day, the first dosing was administered within a few seconds after virus inoculation under anesthesia in the morning of the first day (defined as immediately after virus inoculation). Mice were treated with BXM (0.5 mg/kg/dose [only low dose model], 5 mg/kg/dose, or 50 mg/kg/dose) twice a day (12 hour interval between each dosing) for 1 or 5 day(s) by oral gavage. BXM was suspended with 0.5 w/v% methylcellulose (MC). For the controls, vehicle (0.5 w/v% MC) or oseltamivir phosphate (OSP, 5 mg/kg/dose [clinically-equivalent dose; 75 mg/kg/day^39^] or 50 mg/kg/dose) was administrated twice a day for 5 days by oral gavage. OSP was dissolved with 0.5 w/v% MC. Dosing volume was 10 mL/kg calculated by body weight before each dosing. Survival rates and body weight changes were then monitored through a 21 (high dose infection model) or 28-day (low dose infection model) period after the infection (n = 10/group). Virus titers in the lungs of mice at indicated time points were determined in MDCK cells. The viral RNAs derived from lung homogenates of BXM-treated mice was extracted by PureLink Viral RNA/DNA Mini Kit (Thermo Fisher Scientific) according to the manufacturer’s protocol. Reverse transcription reaction, amplification of cDNA and sequencing reaction were performed as previously reported^64^. Primers used in this study were as follows; PA-1F, 5′-ATATCGTCTCGTATTAGTAGAAACAAGGGTGTTTT-3′ and PA-955R, 5′-TGCATTTGATTGCATCATATAG-3′. Sequence analysis of PA N-terminal domain (the PA gene of A/Anhui/1/2013 [H7N9] strain) was performed by Sanger sequencing method using the 3500/3500xL genetic analyzer (Life Technologies). All animals were housed in self-contained units (Tokiwa Kagaku) at the BSL-3 and ABSL-3 facilities of the Faculty of Veterinary Medicine, Hokkaido University, Japan. Animal experiments were performed according to the guidelines of the institutional animal care and use committee of Hokkaido University (Approval Number 15-0063, 16-0107 and 16-0108). The mice were euthanized when they lost greater than 30% of their body weight compared with their pre-infection weight.

#### Experiment 2

Under anesthesia, mice were infected intranasally with low dose of A/Anhui/1/2013 (H7N9) virus. Mice were treated with BXM (5 or 50 mg/kg/dose) twice a day for 5 days by oral gavage beginning immediately, 24 or 48 hours after virus inoculation. For the controls, vehicle or OSP (5 mg/kg/dose) was administrated twice a day for 5 days by oral gavage beginning immediately after virus inoculation. Dosing volume was 10 mL/kg calculated by body weight before each dosing. Survival rates and body weight changes were then monitored through a 21-day period after the infection (n = 5/group).

### Quantitative analysis of proinflammatory cytokines and chemokines

In experiment 1, levels of proinflammatory cytokines and chemokines, including interleukin (IL)-6, monocyte chemoattractant protein (MCP)-1, macrophage inflammatory protein (MIP)-1α and interferon (IFN)-γ in the lungs, were quantitatively determined using Quantikine ELISA (R&D Systems). The lungs were collected from the virus titer experiments infected with 4.0 × 10^5^ TCID_50_ of A/Anhui/1/2013 (H7N9) strain at 1 (only for 1-day treatment group), 3 and 5 dpi. The collected lungs were homogenized, and each sample was processed according to the manufacturer’s protocol.

### Statistical analysis

For the comparison of the survival time after infection between each BXM-treated group and vehicle-treated or OSP-treated group, the log-rank test was applied in experiment 1 and 2. For the comparison of body weight changes of mice throughout the treatment period, virus titers in the lung tissues or cytokines and chemokines levels in the lung tissues between each BXM-treated group and vehicle-treated or OSP-treated group at each time point were evaluated using Dunnett’s multiple-comparison method in experiment 1. Statistical analysis was performed using the statistical analysis software SAS version 9.2 for Windows (SAS Institute, Cary, NC). *P* values < 0.05 were considered statistically significant.

### Ethics statement

All animals were housed in self-contained units (Tokiwa Kagaku) at the BSL-3 and ABSL-3 facilities of the Faculty of Veterinary Medicine, Hokkaido University, Japan. Animal experiments were performed according to the guidelines of the institutional animal care and use committee of Hokkaido University (Approval Number 15-0063, 16-0107 and 16-0108). The mice were euthanized when they lost greater than 30% of their body weight compared with their pre-infection weight.

## Supplementary information

Dataset 1
