# Supplementary material for: Inhibition of avian-origin influenza A(H7N9) virus by the novel cap-dependent endonuclease inhibitor baloxavir marboxil
Source: Sci Rep. 2019 Mar 5;9:3466. doi: 10.1038/s41598-019-39683-4 (PMC6401108; doi:10.1038/s41598-019-39683-4)
Supplement: Supplementary file 1 — Dataset 1 [file 41598_2019_39683_MOESM1_ESM.docx]

**Supplementary Dataset**

**Inhibition of avian-origin influenza A(H7N9) virus by the novel cap-dependent endonuclease inhibitor baloxavir marboxil**

Keiichi Taniguchi^1, 2^, Yoshinori Ando^1^, Haruaki Nobori^1, 3^, Shinsuke Toba^1, 3^, Takeshi Noshi^1^, Masanori Kobayashi^1^, Makoto Kawai^1^, Ryu Yoshida^1^, Akihiko Sato^1, 3^, Takao Shishido^1^, Akira Naito^1^, Keita Matsuno^2, 4^, Masatoshi Okamatsu^2^, Yoshihiro Sakoda^2, 4^, and Hiroshi Kida^3, 4^

^1^Shionogi & Co., Ltd., Osaka, Japan. ^2^Department of Disease Control, Faculty of Veterinary Medicine, Hokkaido University, Japan. ^3^Research Center for Zoonosis Control, Hokkaido University, Japan. ^4^Global Station for Zoonosis Control, Global Institution for Collaborative Research and Education (GI-CoRE), Hokkaido University, Japan.

(a) A/Anhui/1/2013 (H7N9)

|  | The mean degree of virus reduction ± SD (Log_10_ TCID_50_/mL) | | | | | | | | |
| --- | --- | --- | --- | --- | --- | --- | --- | --- | --- |
| Drug concentration (nM) | Baloxavir acid | | | Oseltamivir acid | | | Favipiravir | | |
| 312500 | NT^a^ | | | NT^a^ | | | -2.69 | ± | 1.27 |
| 62500 | NT^a^ | | | NT^a^ | | | -1.71 | ± | 0.46 |
| 12500 | NT^a^ | | | NT^a^ | | | -0.91 | ± | 0.29 |
| 2500 | NT^a^ | | | -3.71 | ± | 0.18 | -0.67 | ± | 0.21 |
| 500 | NT^a^ | | | -3.17 | ± | 0.15 | -0.42 | ± | 0.15 |
| 100 | -4.97 | ± | 0.52 | -1.95 | ± | 0.39 | -0.29 | ± | 0.04 |
| 20 | -4.61 | ± | 0.25 | -1.25 | ± | 0.19 | -0.24 | ± | 0.20 |
| 4 | -2.83 | ± | 0.55 | -0.43 | ± | 0.16 | -0.19 | ± | 0.19 |
| 0.8 | -1.13 | ± | 0.62 | -0.29 | ± | 0.01 | NT^a^ | | |
| 0.16 | -0.11 | ± | 0.16 | NT^a^ | | | NT^a^ | | |
| 0.032 | -0.28 | ± | 0.10 | NT^a^ | | | NT^a^ | | |
|  |  |  |  |  |  |  |  |  |  |
|  | The mean degree of virus reduction ± SD  (Log_10_ TCID_50_/mL) | | | | | |  |  |  |
| Drug concentration (nM) | Zanamivir hydrate | | | Laninamivir | | |  |  |  |
| 2000 | -3.46 | ± | 0.08 | -3.88 | ± | 0.46 |  |  |  |
| 400 | -2.74 | ± | 0.73 | -3.24 | ± | 0.34 |  |  |  |
| 80 | -2.18 | ± | 0.75 | -2.65 | ± | 0.35 |  |  |  |
| 16 | -0.79 | ± | 0.08 | -1.25 | ± | 0.25 |  |  |  |
| 3.2 | -0.26 | ± | 0.13 | -0.20 | ± | 0.13 |  |  |  |
| 0.64 | -0.08 | ± | 0.16 | 0.04 | ± | 0.25 |  |  |  |

**Supplementary Table 1. Antiviral activities of several concentrations of BXA and reference compounds against human and avian A(H7N9) viruses in a yield reduction assay in MDCK cells.** Experimental protocols were the same as in Table 2. a; not tested. Data represent the mean degree of virus reduction ± standard deviation from three independent experiments with in MDCK cells.

(b) A/Anhui/1/2013 NA-R292K (H7N9)

|  | The mean degree of virus reduction ± SD  (Log_10_ TCID_50_/mL) | | |
| --- | --- | --- | --- |
| Drug concentration (nM) | Baloxavir acid | | |
| 100 | -2.24 | ± | 0.31 |
| 20 | -2.19 | ± | 0.41 |
| 4 | -1.55 | ± | 0.25 |
| 0.8 | -0.91 | ± | 0.23 |
| 0.16 | -0.24 | ± | 0.25 |
| 0.032 | -0.07 | ± | 0.36 |
|  |  |  |  |
|  | The mean degree of virus reduction ± SD  (Log_10_ TCID_50_/mL) | | |
| Drug concentration (nM) | Oseltamivir acid | | |
| 250000 | -1.29 | ± | 0.04 |
| 50000 | -0.46 | ± | 0.12 |
| 10000 | -0.07 | ± | 0.37 |
| 2000 | -0.13 | ± | 0.26 |
| 400 | 0.06 | ± | 0.09 |
| 80 | -0.10 | ± | 0.20 |
|  |  |  |  |
|  | The mean degree of virus reduction ± SD  (Log_10_ TCID_50_/mL) | | |
| Drug concentration (nM) | Favipiravir | | |
| 500000 | -1.80 | ± | 0.19 |
| 100000 | -1.47 | ± | 0.12 |
| 20000 | -1.10 | ± | 0.15 |
| 4000 | -0.38 | ± | 0.06 |
| 800 | -0.25 | ± | 0.25 |
| 160 | 0.00 | ± | 0.03 |

**Supplementary Table 1. (continued)**

(c) A/duck/Japan/AQ-HE28-3/2016 (H7N9)

|  | The mean degree of virus reduction ± SD (Log_10_ TCID_50_/mL) | | | | | | | | |
| --- | --- | --- | --- | --- | --- | --- | --- | --- | --- |
| Drug concentration (nM) | Baloxavir acid | | | Oseltamivir acid | | | Favipiravir | | |
| 312500 | NT^a^ | | | NT^a^ | | | -1.63 | ± | 0.15 |
| 62500 | NT^a^ | | | NT^a^ | | | -1.39 | ± | 0.09 |
| 12500 | NT^a^ | | | NT^a^ | | | -1.08 | ± | 0.20 |
| 2500 | NT^a^ | | | -1.65 | ± | 0.15 | -0.22 | ± | 0.55 |
| 500 | NT^a^ | | | -1.40 | ± | 0.23 | -0.13 | ± | 0.31 |
| 100 | -1.71 | ± | 0.18 | -1.29 | ± | 0.19 | -0.13 | ± | 0.29 |
| 20 | -1.71 | ± | 0.18 | -0.88 | ± | 0.07 | -0.11 | ± | 0.32 |
| 4 | -1.60 | ± | 0.23 | -0.07 | ± | 0.25 | -0.01 | ± | 0.30 |
| 0.8 | -1.19 | ± | 0.14 | -0.20 | ± | 0.14 | NT^a^ | | |
| 0.16 | -0.03 | ± | 0.21 | NT^a^ | | | NT^a^ | | |
| 0.032 | -0.01 | ± | 0.16 | NT^a^ | | | NT^a^ | | |

(d) A/duck/Japan/AQ-HE29-22/2017 (H7N9)

|  | The mean degree of virus reduction ± SD (Log_10_ TCID_50_/mL) | | | | | | | | |
| --- | --- | --- | --- | --- | --- | --- | --- | --- | --- |
| Drug concentration (nM) | S-033447 | | | Oseltamivir acid | | | Favipiravir | | |
| 312500 | NT^a^ | | | NT^a^ | | | -2.42 | ± | 0.40 |
| 62500 | NT^a^ | | | NT^a^ | | | -1.55 | ± | 0.30 |
| 12500 | NT^a^ | | | NT^a^ | | | -0.99 | ± | 0.28 |
| 2500 | NT^a^ | | | -2.45 | ± | 0.15 | -0.31 | ± | 0.09 |
| 500 | NT^a^ | | | -2.22 | ± | 0.23 | 0.05 | ± | 0.15 |
| 100 | -2.53 | ± | 0.28 | -1.76 | ± | 0.19 | 0.14 | ± | 0.35 |
| 20 | -2.51 | ± | 0.23 | -1.27 | ± | 0.30 | -0.03 | ± | 0.45 |
| 4 | -2.28 | ± | 0.41 | -0.63 | ± | 0.19 | -0.26 | ± | 0.33 |
| 0.8 | -1.02 | ± | 0.13 | -0.16 | ± | 0.18 | NT^a^ | | |
| 0.16 | -0.39 | ± | 0.44 | NT^a^ | | | NT^a^ | | |
| 0.032 | -0.13 | ± | 0.43 | NT^a^ | | | NT^a^ | | |

**Supplementary Table 1. (continued)**

(e) A/duck/Japan/AQ-HE30-1/2018 (H7N3)

|  | The mean degree of virus reduction ± SD (Log_10_ TCID_50_/mL) | | | | | | | | |
| --- | --- | --- | --- | --- | --- | --- | --- | --- | --- |
| Drug concentration (nM) | S-033447 | | | Oseltamivir acid | | | Favipiravir | | |
| 312500 | NT^a^ | | | NT^a^ | | | -1.69 | ± | 0.31 |
| 62500 | NT^a^ | | | NT^a^ | | | -1.50 | ± | 0.25 |
| 12500 | NT^a^ | | | NT^a^ | | | -0.88 | ± | 0.23 |
| 2500 | NT^a^ | | | -1.69 | ± | 0.28 | -0.42 | ± | 0.40 |
| 500 | NT^a^ | | | -1.56 | ± | 0.32 | 0.00 | ± | 0.48 |
| 100 | -1.75 | ± | 0.23 | -1.39 | ± | 0.27 | 0.00 | ± | 0.45 |
| 20 | -1.75 | ± | 0.23 | -1.03 | ± | 0.26 | 0.09 | ± | 0.47 |
| 4 | -1.61 | ± | 0.20 | -0.31 | ± | 0.32 | -0.03 | ± | 0.23 |
| 0.8 | -0.92 | ± | 0.41 | -0.03 | ± | 0.25 | NT^a^ | | |
| 0.16 | 0.21 | ± | 0.32 | NT^a^ | | | NT^a^ | | |
| 0.032 | 0.29 | ± | 0.26 | NT^a^ | | | NT^a^ | | |

**Supplementary Table 1. (continued)**


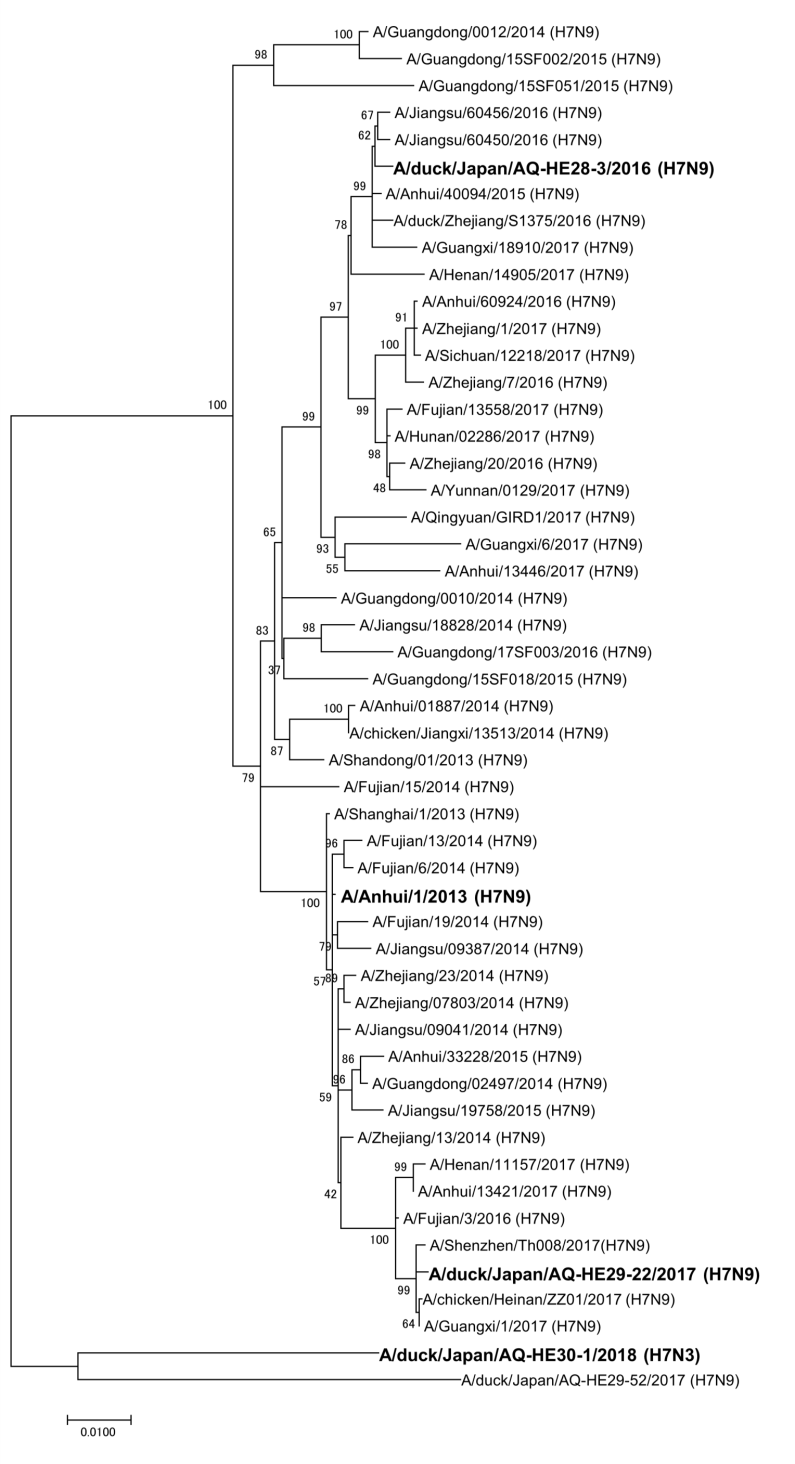


**Supplementary Figure 1. Phylogenetic tree of the PA gene segments of A(H7N9) viruses.** The nucleotide sequences of the H7 PA genes were analyzed by the maximum-likelihood method along with the corresponding genes of reference strains using MEGA 7.0 software (<http://www.megasoftware.net/>). The horizontal distances are proportional to the minimum number of nucleotide differences required to join nodes and sequences. Numbers at the nodes indicate confidence levels in a bootstrap analysis with 1000 replications. The viruses employed in the present study are highlighted in boldface. The dataset of reference viruses is modified from Shibata *et al* ^45^.


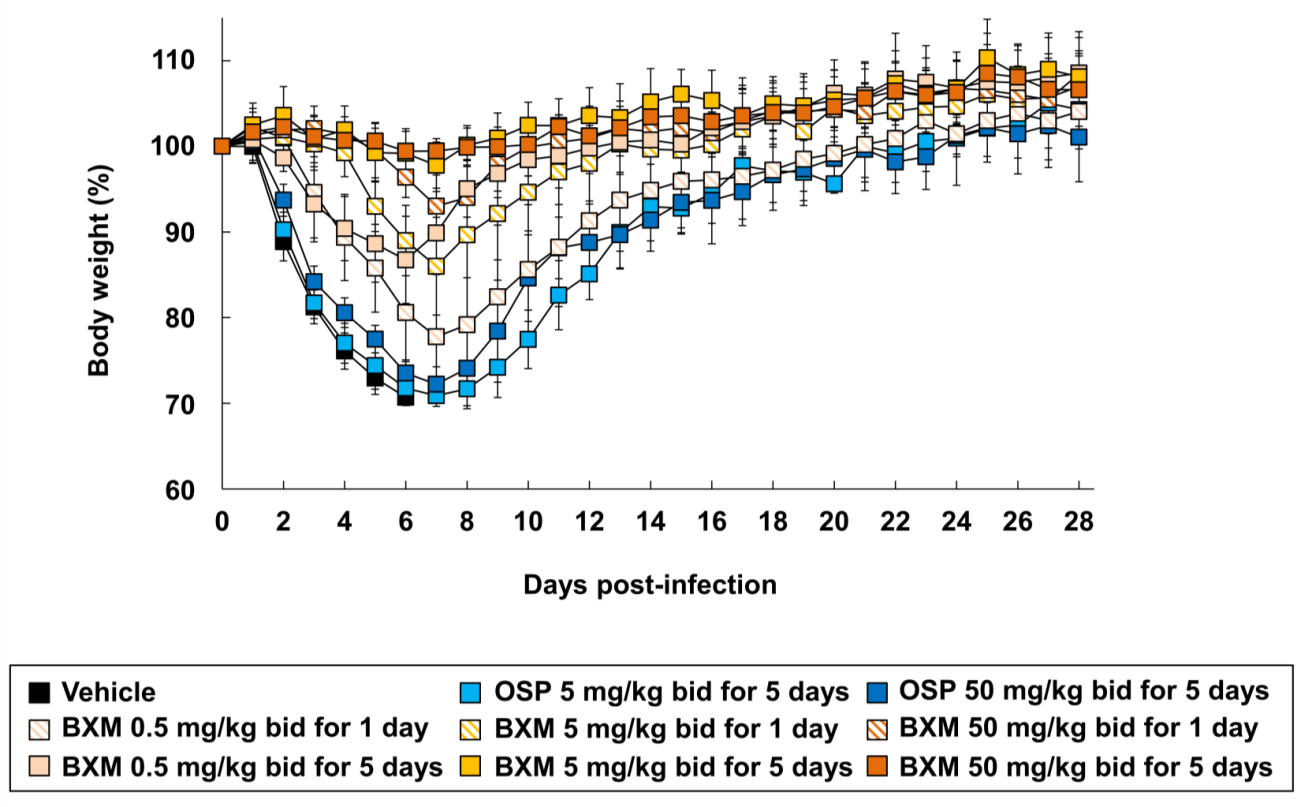


**Supplementary Figure 2. Therapeutic effects of BXM on body weight change in mice infected with a low dose of the A(H7N9) virus.** Mice were treated as described in the legend to Fig. 2. Body weight at day 0 was set as 100% and the ratio (%) of body weight was calculated. bid (bis in die): twice a day.


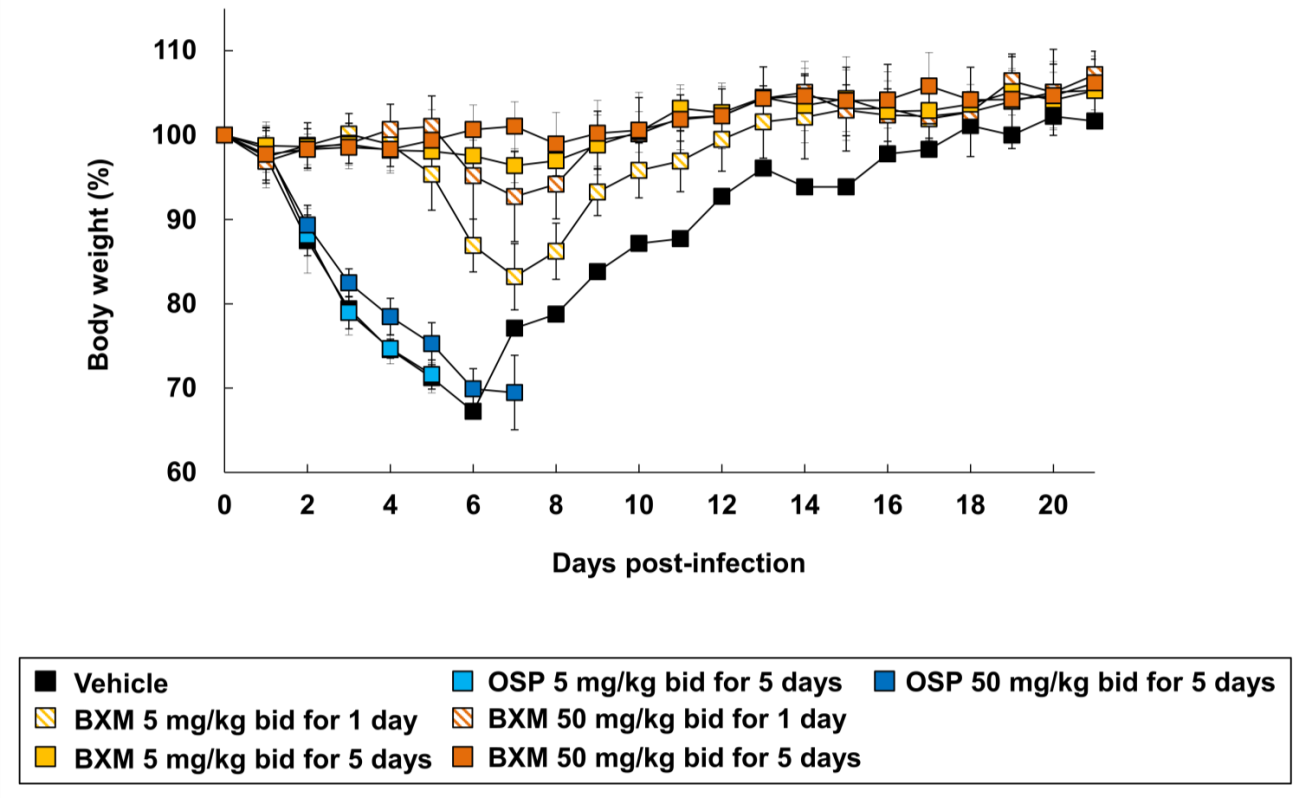


**Supplementary Figure 3. Therapeutic effects of BXM on body weight change in mice infected with a high dose of the A(H7N9) virus.** Mice were treated as described in the legend to Fig. 5. Body weight at day 0 was set as 100% and the ratio (%) of body weight was calculated. bid (bis in die): twice a day.
